# Implementation of a loss-of-function system to determine growth and stress-associated mutagenesis in *Bacillus subtilis*

**DOI:** 10.1371/journal.pone.0179625

**Published:** 2017-07-11

**Authors:** Norberto Villegas-Negrete, Eduardo A. Robleto, Armando Obregón-Herrera, Ronald E. Yasbin, Mario Pedraza-Reyes

**Affiliations:** 1 Department of Biology, Division of Natural and Exact Sciences, University of Guanajuato, Guanajuato, Mexico; 2 School of Life Sciences, University of Nevada, Las Vegas, Nevada, United States of America; 3 College of Arts and Sciences, University of Missouri—St. Louis, St. Louis, Missouri, United States of America; National Center for Biotechnology Information, UNITED STATES

## Abstract

A forward mutagenesis system based on the acquisition of mutations that inactivate the thymidylate synthase gene (TMS) and confer a trimethoprim resistant (Tmp^r^) phenotype was developed and utilized to study transcription-mediated mutagenesis (TMM). In addition to *thyA*, *Bacillus subtilis* possesses *thyB*, whose expression occurs under conditions of cell stress; therefore, we generated a *thyB*^-^ *thyA*^+^ mutant strain. Tmp^r^ colonies of this strain were produced with a spontaneous mutation frequency of ~1.4 × 10^−9^. Genetic disruption of the canonical mismatch (MMR) and guanine oxidized (GO) repair pathways increased the Tmp^r^ frequency of mutation by ~2–3 orders of magnitude. A wide spectrum of base substitutions as well as insertion and deletions in the ORF of *thyA* were found to confer a Tmp^r^ phenotype. Stationary-phase-associated mutagenesis (SPM) assays revealed that colonies with a Tmp^r^ phenotype, accumulated over a period of ten days with a frequency of ~ 60 ×10^−7^. The Tmp^r^ system was further modified to study TMM by constructing a Δ*thyA* Δ*thyB* strain carrying an IPTG-inducible P*spac-thyA* cassette. In conditions of transcriptional induction of *thyA*, the generation of Tmp^r^ colonies increased ~3-fold compared to conditions of transcriptional repression. Further, the Mfd and GreA factors were necessary for the generation of Tmp^r^ colonies in the presence of IPTG in *B*. *subtilis*. Because GreA and Mfd facilitate transcription-coupled repair, our results suggest that TMM is a mechanim to produce genetic diversity in highly transcribed regions in growth-limited *B*. *subtilis* cells.

## Introduction

The ability of stressed microbial subpopulations to acquire genetic alterations in response to a persistent non-lethal pressure allowing them to escape from growth-limiting conditions has been termed adaptive or stationary-phase mutagenesis (SPM) [1–3]. This phenomenon promotes genetic diversity and is conserved in prokaryotes and eukaryotes [4–7].

In *Bacillus subtilis*, the mechanisms underlying SPM have been successfully investigated in the strain YB955 bearing the chromosomal auxotrophies *hisC952*, *leuC427* and *metB5* [6]. Using this gain-of-function (reversion) mutagenesis system it has been shown that adaptive mutations arise from error-prone processing of mismatched and chemically modified DNA bases [8–11]. Additional evidence revealed a direct correlation between transcriptional derepression and SPM in non-dividing *B*. *subtilis* cells [12, 13]. Recent results showed that in growth-limited *B*. *subtilis* cells, the transcription repair-coupling factor (Mfd) promotes mutagenic events in transcriptionally active genes coordinating error-prone repair events that required nucleotide excision (NER) and base excision (BER) repair components as well as low-fidelity DNA synthesis [14]. Notably, a mutagenic pathway dependent on Mfd, the NER system and the error prone polymerase PolY1 that prevents conflicts between the replicative and transcriptional machineries has been recently described in growing *B*. *subtilis* cells [15]. In *Escherichia coli*, the elongation factor of the RNA polymerase NusA has been found to be necessary for stress-induced mutagenesis [16]. In addition to Mfd, *B*. *subtilis* possesses the transcriptional factors NusA and GreA [17, 18]; however, a possible contribution of these factors in modulating transcriptional-mediated mutagenic events in nutritionally stressed, non-growing *B*. *subtilis*, remains to be elucidated.

Two types of genetic alterations affect organism's physiology, namely, the gain-of- (i.e., reversion mutagenesis) and the loss-of-function (i.e., forward mutagenesis) mutations; the former may generate a product with an enhanced or a novel function whereas the latter one leads to reducing or abolishing protein function [19]. As noted above, SPM frequencies in *B*. *subtilis* have commonly calculated from mutation events occurring in strain YB955 containing point mutations in the chromosomal genes, *metB5* (ochre), *leuC427* (missense) and *hisC952* (amber) [6]. However, evolutionary experiments conducted in distinct microorganisms have revealed that loss-of- or modification-of- are by far more frequent than gain-of-function mutational events [19].

Thymidine synthesis plays an essential role in DNA metabolism. In both, prokaryotes and eukaryotes, thymidylate synthase (TMS) converts dUMP to dTMP using *N*^*5*^*N*^*10*^*-*methylenetetrahydrofolate as cofactor [20]. Thus, tetrahydrofolate analogs such as aminopterin or trimethoprim that inhibit dihydrofolate reductase (DHFR) also inhibit thymidine synthesis [21, 22]. *B*. *subtilis* possesses *thyA* and *thyB* encoding TMSs [23, 24]. In this bacterium, thymine auxotrophs incorporate this metabolite much more efficiently than the wild type strain and are able to grow in the presence of aminopterin or trimethoprim [20–22]. Thus, loss of TMS function allows selection of Tmp^r^ mutants that requires exogenous thymine for growth [20–22].

Here, we developed a loss-of-function mutagenesis system based on the production of trimethoprim resistant colonies (Tmp^r^) resulting from mutations that inactivate TMS as an efficient and more direct method to analyze growth and SPM in *B*. *subtilis*. The use of this system showed that a null *thyB B*. *subtilis* strain proficient for *thyA* accumulated a high frequency of adaptive Tmp^r^ colonies. Furthermore, under growth-limited conditions, derepression of a wild type *thyA* gene in a Δ*thyA* Δt*hyB* background revealed a positive correlation between transcription and accumulation of Tmp^r^ colonies. Interestingly, the generation of transcription-associated Tmp^r^ mutants was dependent not only on Mfd but also on GreA, two proteins known to process RNA polymerase (RNAP) pausing. Thus, our results suggest that under conditions of nutritional stress RNAP backtracking and/or RNAP pausing promote mutagenesis in non-growing *B*. *subtilis* cells.

## Materials and methods

### Bacterial strains and culture conditions

The bacterial strains used in this study are listed in Table 1. *B*. *subtilis* YB955 is a prophage-“cured” strain that contains the *hisC952*, *metB5*, and *leuC427* alleles [6, 25]. The procedures for transformation and isolation of chromosomal and plasmid DNA were as described previously [26–28]. Liquid cultures of *B*. *subtilis* strains were routinely grown in Penassay broth (PAB) (antibiotic A3 medium; Difco Laboratories, Sparks, MD). When required, neomycin (Neo; 10 μg ml^-1^); tetracycline (Tet; 10 μg ml^-1^); spectinomycin (Sp; 100 μg ml^-1^); erythromycin (Em; 1 μg ml^-1^ or 5 μg ml^-1^); chloramphenicol (Cm; 5 μg ml^-1^); kanamycin (Kan; 50 μg ml^-1^); trimethoprim (Tmp; 10 μg ml^-1^), or IPTG (0.07 mM) was added to the medium. *E*. *coli* cultures were grown in Luria-Bertani (LB) medium supplemented with ampicillin to a final concentration of 100 μg ml^-1^. The PCR products were generated with Vent DNA polymerase (New England BioLabs, Ipswich, MA) and the set of homologous oligonucleotide primers described in Table 2.

**Table 1:** Strains and plasmid used in this study.

Strain or Plasmid	Genotype or description^*a*^	Construction or source^*c*^
***B*. *subtilis***		
YB955	*hisC952 metB5 leuC 427 xin-1 sp*_*SENS*	[6]
PERM573	YB955 Δ*ytkD*::*neo* Δ*mutM*::*tet* Δ*mutY*::*sp* Neo^r^ Spc^r^ Tet^r^	[9]
PERM739	YB955 Δ*mutSL*::*neo* Neo^r^	[10]
YB9800	YB955 Δ*mfd*::*tet* Tet^r^	[12]
PERM1000^*b*^	YB955 Δ*thyA*::*neo* Neo^r^	This study
PERM1037^*b*^	YB955 Δ*thyB*::*em* Em^r^	pPERM1013→YB955
PERM1074^*b*^	YB955 Δ*thyA*::*neo* Δ*thyB*::*em* Neo^r^ Em^r^	pPERM1013→PERM1000^*a*^
PERM1075^*b*^	YB955 Δ*thyA*::*neo* Δ*thyB*::*em* with a P*hs* construct inserted into *amyE* locus; Neo^r^ Em^r^ Sp^r^	This study
PERM1100^*b*^	YB955 Δ*thyA*::*neo* Δ*thyB*::*em* with a P*hs*-*thyA* construct inserted into *amyE* locus; Neo^r^ Em^r^ Sp^r^	pPERM1099→PERM1074^*a*^
PERM1171^b^	YB955 Δ*thyA*::*neo* Δ*thyB*::*em* Δ*mfd*::*tet* with a P*hs*-*thyA* construct inserted into *amyE* locus; Neo^r^ Em^r^ Tc^r^ Sp^r^	YB9800 → PERM1100
PERM1192^*b*^	YB955 Δ*thyA*::*neo* Δ*thyB*::*em* Δ*greA*::*cm* with a P*hs*-*thyA* construct inserted into *amyE* locus; Neo^r^ Em^r^ Cm^r^ Sp^r^	pPERM1191 →PERM1171
PERM1491	YB955 Δ*thyB*::*em* Δ*ytkD*::*neo* Δ*mutM*::*tet* Δ*mutY*::*sp* Em^r^ Neo^r^ Tet^r^ Sp^r^	PERM573 → PERM1037
PERM1565	YB955 Δ*thyB*::*em* Δ*mutSL*::*neo* Em^r^ Neo^r^	PERM153 → PERM1037
**Plasmids**		
pBEST	PGEM4 containing the neomycin resistance cassette under control of *repU* promoter of *B*. *subtilis*	[28]
pdr-111-*amyE*-Phyperspank	*bla spec* carrying Phyperspank promoter	[13]
pMUTIN4	Integrational *lacZ* fusion vector; Amp^r^ Em^r^	[29]
pMUTIN4-Cat	pMUTIN4-*cat*; Cm^r^	[11]
pPERM1013	pMUTIN4 with a 357-bp HindIII/BamHI PCR product from inside of the 5´ end of *thyB* ORF; Amp^r^	This study
pPERM1014	pBEST with a 493-bp BamHI/SacI PCR product from the 5´ end of *thyA* and with a 663-bp BamHI/SacI PCR product from the 3´ end of *thyA* ORF; Amp^r^	This study
pPERM1098	pJET1.2/blunt carrying a 1,194-bp SalI/SphI PCR fragment from *thyA* ORF; Amp^r^	This study
pPERM1099	pdr-111-amyE-Phyperspank carrying a 1,194-bp SalI/SphI fragment from pPERM1098; Sp^r^	This study
pPERM1191	pMUTIN4 with a 222-bp EcoRI/BamHI PCR fragment from *greA* ORF; Cm^r^	This study

^***a***^Em, Erithromycin; Amp, Ampicillin; Neo, Neomycin; Sp, Spectinomycin; Tet, Tetracycline; Cm, Chloramphenicol.

^*b*^”X”→”Y” indicates that “strain Y” was transformed with DNA from “source X”.

^*c*^The background for this strain is YB955.

**Table 2:** Oligonucleotide primers employed in the PCR reactions.

^a^Oligonucleotide	(Sequence 5' to 3')	Restriction site (underlined)
1) *thyA* FW	CGGCATGCGGTATACACAAACACCTGAC	SphI
2) *thyA* RV	GCGTCGACGGAACCTCTGAGTTGTC	SalI
3) *thyA* FW	CGGGATCCCGCATGATTGCTCAAGTGAC	BamHI
4) *thyA* RV	GCGAGCTCCAAATGCGTTGATACCTGCTG	SalI
5) *thyB* FW	CGAAGCTTGAGCATGGTGAGAAAAAGG	HindIII
6) *thyB* RV	GCGGATCCGCTGGACGATTAAGCGTCTG	BamHI
7) ORF *thyA* FW	GCGTCGACGCAACTGAAAATGAAAGAAGG	SalI
8) ORF *thyA* RV	GCGCATGCATAAGGGTGGAAATTGGTATG	SphI
9) *greA* FW	GCGAATTCGCGAGAAGCTTCGGAGACC	EcoRI
10) *greA* RV	GCGGATCCCGCGCTTCCGACGATCGTA	BamHI
11) *thyA*RT FW	GGGACTCAGATGGAACACCG	-
12) *thyA*RT RV	CCAAATATGGACGCCCATC	-
13) *veg* FW	TGGCGAAGACGTTGTCCGATATTA	-
14) *veg* RV	CGGCCACCGTTTGCTTTTAAC	-

^a^F, forward; R, Reverse

### Strains construction

Knockouts in the genes of interest were constructed by marker exchange between the chromosome- and plasmid-borne alleles. A plasmid to disrupt *thyA* was constructed as follows, a 493-bp fragment from the 5'-*thyA* region (-300 to +164 relative to *thyA* start codon) and a 473-bp fragment from the 3'-*thyA* region (+600 to +1073 relative to *thyA* start codon) were PCR amplified using chromosomal DNA from *B*. *subtilis* 168. The set of oligonucleotide primers 3, 4 and 5, 6 (Table 2) were used for amplification of the 5'-*thyA* and 3'-*thyA* fragments, respectively. The amplified 5'-*thyA* and 3'-*thyA* fragments were cloned between the SphI/SalI and BamHI/SalI sites of pBEST502 [29]. The resulting construction pPERM1014 was replicated in *E*. *coli* XL10-GOLD Kan^r^ (Stratagene, Cedar Creek, TX) and employed to transform the strain *B*. *subtilis* YB955 to neomycin resistance, thus generating strain *B*. *subtilis* PERM1000 (Table 1).

A Δ*thyB* strain was generated by amplifying a fragment from position 38 to 675 from the *thyB* open reading frame was amplified by PCR using chromosomal DNA from *B*. *subtilis* YB955 and the oligonucleotide primers 5 and 6 (Table 2). The PCR product was cloned between the HindIII and BamHI sites of the integrative plasmid pMUTIN4 [30]. The resulting construct was designated pPERM1013 and used to transform *B*. *subtilis* YB955 and PERM1000, thus generating strains PERM1037 (Δ*thyB*; Em^r^) and PERM1074 (Δ*thyA* Δ*thyB*; Neo^r^ Em^r^), respectively (Table 1).

Genetic inactivation of error prevention GO system (*ytkD*, *mutM* and *yfhQ*) from *B*. *subtilis* YB955 was achieved by transforming the strain Δ*thyB* (PERM1037; Table 1) with genomic DNA isolated from *B*. *subtilis* PERM573 (Δ*ytkD*::Neo Δ*mutM*::Tet Δ*mutY*::Spc). This procedure generated the strain *B*. *subtilis* PERM1491 (Δ*thyB* GO; Em^r^ Cm^r^ Tet^r^ Spc^r^), respectively (Table 1). The *thyB mutSL* mutant in the YB955 background was generated by transforming strain PERM151 (Δ*mutSL*::Neo) [8] with genomic DNA isolated from *B*. *subtilis* PERM1037 (Δ*thyB*), thus generating strain PERM1565 (Δ*thyB mutSL*; Em^r^ Neo^r^) (Table 1). Disruptions of the appropriate chromosomal genes were confirmed by PCR.

The strain containing the *thyA* gene under the control of an IPTG-inducible promoter was constructed as follows. The open reading frame (ORF) of *thyA* was PCR amplified from *B*. *subtilis* 168 chromosomal DNA and the oligonucleotide primers 7 and 8 (Table 2). The PCR product (1,194 bp) was purified from a low-melting-point agarose gel and cloned between the SalI and SphI sites of the *amyE* integrative vector pdr111 (a gift from David Rudner), immediately downstream of the IPTG-inducible Phyperspank promoter (P*hs*). The resulting plasmid (pPERM1099) was replicated in *E*. *coli* XL10-GOLD (Stratagene). This construct was transformed and integrated into the *amyE* locus of *B*. *subtilis* PERM1074 (Δ*thyA thyB*) to generate the strain *B*. *subtilis* PERM1100 (Δ*thyA* Δ*thyB*; *amyE*::P*hs*-*thyA*; Neo^r^ Em^r^ Spc^r^) (Table 1). As an experimental control, the empty pdr111 vector was also recombined in the *amyE* locus thus generating the strain *B*. *subtilis* PERM1075 (Δ*thyA* Δ*thyB*; *amyE*::P*hs*; Neo^r^ Em^r^ Spc^r^) (Table 1).

To disrupt *mfd*, competent cells of strain *B*. *subtilis* PERM1100 were transformed with chromosomal DNA of *B*. *subtilis* YB9800 (Δ*mfd*::Cm) [12] to generate the strain *B*. *subtilis* PERM1171 (Δ*thyA* Δ*thyB* Δ*mfd*; *amyE*::P*hs*-*thyA*; Neo^r^ Em^r^ Cm^r^ Spc^r^) (Table 1).

An integrative plasmid designed to inactivate *greA* was constructed as follows. A fragment of *greA* was first amplified by PCR using chromosomal DNA from *B*. *subtilis* 168 and the set of oligonucleotide primers 9 and 10 (Table 2). The 222-bp PCR product, extending from position 115-bp to 318-bp downstream of the *greA* start codon was cloned between the EcoRI-BamHI sites of the integrative plasmid pMUTIN4-Cat [11]. The resulting construct designated pPERM1191was used to transform *B*. *subtilis* PERM1100, generating strain PERM1192 (Δ*thyA* Δ*thyB* Δ*greA*; *amyE*::P*hs*-*thyA*; Neo^r^ Em^r^ Cm^r^ Spc^r^) (Table 1). The single- or double-crossover events leading to inactivation of the appropriate genes and integration of transcriptional cassettes were corroborated by PCR with specific oligonucleotide primers.

### Stationary-phase mutagenesis soft-agar overlay assays

Cultures were grown in flasks containing antibiotic A3 medium with aeration (250 rpm) at 37°C to 90 min after the cessation of exponential growth (designated T_*90*_). Growth was monitored with a spectrophotometer measuring the optical density at 600nm (OD_600_). The cultures were centrifuged at 10, 000 × *g* for 10 min and resuspended in 10 ml of 1X Spizizen Minimal Salts (SMS) [31]. Aliquots of 0.1 ml were then spread plated on Spizizen minimal medium (SMM) containing 1X SMS, 0.5% (w/v) dextrose, 50 μg isoleucine ml^-1^, 50 μg glutamate ml^-1^, 1.5% (w/v) agar (BD, Bioxom), 50 μg histidine ml^-1^ of and a trace amount (200 ng ml^-1^) of leucine and methionine (amino acids from Sigma-Aldrich, St. Louis, MO), with or without 0.07 mM IPTG. The initial titer was determined from the 10-ml culture. Starting from 48 h of incubation, a set of plates was overlaid with soft agar (0.7% (w/v) agar and prewarmed at 42°C lacking histidine and containing 0.07 mM IPTG, 50 μg thymine ml^-1^, 10 μg Tmp ml^-1^, 50 μg methionine and leucine ml^-1^. Of note, adjustment of the final concentrations for IPTG, methionine and leucine considered the volume and IPTG concentration in the medium dispensed previous to performing the overlay. The plates were incubated for two days, and the number of Tmp^r^ colonies was scored. The initial titers were used to normalize the cumulative number of resistant colonies per day to the number of CFU plated. Assays were replicated three times, and the experiment was repeated at least twice. The viability of the non-revertant cell background was assessed every other day as follows. Using a sterile Pasteur pipette, a plug of agar was removed from the non-revertant background of each of five plates corresponding to one type of selection (no leucine and methionine without IPTG or no leucine and methionine with IPTG). The plugs were combined in 0.4 ml of 1X SMS, serially diluted, and plated in triplicate on SMM containing 50 μg ml^-1^ of the required amino acids. Colonies were counted after 48 h of incubation.

### Phenotypic analysis of *B*. *subtilis thy* mutants

Trimethoprim resistance of the strains of interest was analyzed on solid SMM containing 1X SMS, 0.5% (w/v) dextrose, 50 μg ml^-1^ isoleucine, glutamate, histidine, methionine, leucine and thymine. All plates contained thymine at 50 μg ml^-1^ and the concentration (μg/ml) of trimethoprim indicated in Table 3. When necessary, the media was supplemented with IPTG to a final concentration of 0.1 mM. Growth was scored after 24 hr at 37°.

**Table 3:** Phenotypes of *B*. *subtilis thy* mutants.

Strain	Genotype	^a^Resistance to Trimethoprim 10 μg ml^-1^
YB955	*thyA*^+^*thyB*^+^	–
PERM1000	*thyA*^-^*thyB*^+^	–
PERM1037	*thyA*^+^*thyB*^-^	–
PERM1074	*thyA*^-^*thyB*^-^	++
^b^PERM1100	*thyA*^-^*thyB*^-^; *amyE*:: P*hs*-*thyA*	_–

^a^Strains were grown on solid Spizizen Minimal Medium (SMM) as described in Materials and Methods. ++, full growth; +, weak growth; -, no growth.

^b^ For this strain, solid SMM was supplemented with 0.1mM IPTG.

### Analysis of mutation frequencies to Tmp^r^ and Rif^r^

Essentially, the appropriate strains were propagated for 12 h at 37^°^C in A3 medium with proper antibiotics. For Tmp^r^, mutation frequencies were determined by plating aliquots on six LB plates containing 10 μg ml^-1^ trimethoprim and 50 μg ml^-1^ thymidine, and the trimethoprim-resistant (Tmp^r^) colonies were counted after 2 days of incubation at 37°C. The same procedure was employed to determine the Rif^r^ phenotype, except that mutant colonies were selected in LB plates containing 10 μg ml^-1^ rifampin. The number of cells used to calculate the frequency of mutation to Tmp^r^ or Rif^r^ was determined by plating aliquots of appropriate dilutions on LB plates without antibiotics and incubating the plates for 24 to 48 h at 37°C. These experiments were repeated at least three times.

### Total RNA extraction

Cultures were grown to saturation in 1X SMS containing 0.5% (w/v) dextrose; 50 μg ml^-1^ of Ile, Glu, methionine, histidine, and leucine; Ho-Le trace elements; 5 mM MgSO_4_; and 1 mM IPTG. Samples were removed at mid-exponential (approximately OD_600nm_ = 0.5) and stationary (150 min after onset of the stationary phase; T_150_) growth phases, centrifuged at 5, 000 × *g* and 4°C for 10 min and frozen at -20°C. RNA was extracted from the pellets using a Tri reagent (Molecular Research Center, Inc. Cincinnati, OH). After DNAse I treatment, the samples were analyzed by PCR to confirm the absence of genomic DNA with Vent DNA polymerase and the set of primers 11 and 12 described in Table 2. The RNA content in the samples was quantitated using a Nanodrop spectrophotometer (Thermo Fisher Scientific, Dubuque, IA).

### Reverse transcription and quantitative real-time PCR

One microgram of RNA was reverse transcribed using an ImPromII reverse transcriptase kit (Promega), as directed, with random hexamers (0.5-μg final concentration). No-reverse transcriptase (NRT) controls were included for all examples. Master mixes for real-time PCR contained Absolute QPCR SYBR green Mix (Thermo Fisher Scientific) and a 70 mM final concentration of the oligonucleotide primers *thyA*RT FW and *thyA*RT RV (191-bp amplicon) or of *veg* FW and veg RV (82-bp amplicon) described in Table 2. Three replicates from each culture condition containing 4 μl of cDNA were assayed and normalized to the expression of the internal control gene *veg* [32, 33]. Two replicates were assessed for NRT and no-template controls. Quantitative real-time PCR was run on a Bio-Rad iCycler iQ Real-Time PCR Detection System (Bio-Rad, Hercules, CA), using the manufacturer´s suggested protocol and an annealing temperature of 57°C, followed by a melting profile and assessment of amplicon size on an agarose gel. Results were calculated by the 2^-ΔΔC(*T)*^ (where C_*T*_ is threshold cycle) method for relative fold expression [34].

### DNA sequencing

Colonies with a Tmp^r^ phenotype were independently propagated in liquid A3 medium supplemented with 10 μg/ml trimethoprim and 50 μg ml^-1^ thymidine and subjected to DNA isolation [27]. The *thyA* open reading frame of each colony was amplified by PCR using high fidelity and specific oligonucleotide primers (Table 2). Sequencing services were carry out by Functional Biosciences, Inc. (Madison, WI).

## Results and discussion

### Spontaneous mutation frequencies to trimethoprim resistance in growing *B*. *subtilis* cells

To implement a loss-of function system to study mutagenesis in *B*. *subtilis*, the *thyA* gene that codes for TMS was considered as a possible target [23, 24]. In *B*. *subtilis* the presence of ThyA prevents the incorporation of exogenous thymine into DNA synthesis [20]; therefore, strains deficient for this activity incorporate this metabolite more efficiently than ThyA-proficient strains [20]. Of note, *thyA* mutants of *B*. *subtilis* are able to grow in medium supplemented with DHFR inhibitors such as trimethoprim; if thymine is present in the culture medium [20], spontaneous colonies with a Tmp^r^ phenotype can be selected [20, 24, 35]. This phenomenon has been described in several bacteria, including *Escherichia coli* [35–37]. *B*. *subtilis* possesses two TMS encoding genes; i.e., *thyA* and *thyB* respectively [20]. It has been reported that *thyB* expression takes place when *B*. *subtilis* is grown under temperatures below 37^°^C contributing with only 5–8% of the total TMS activity present in this bacterium [23]. Since *thyB* is the first cistron of the *thyB*-*dfrA*-*ypkP* operon, we employed a gene construct (pPERM1013; Table 1) to only disrupt *thyB* and left *dfrA-ypkP* under control of the IPTG-inducible P*spac* promoter. Under our experimental conditions, no significant differences were observed in the growth rates of this mutant with respect to those of the parental strain YB955. Thus, in A3 medium, in the absence or presence of 0.1 mM IPTG the *thyB* strain grew with similar doubling time values as those of its parental strain (i.e., 31 ± 2, 30 ± 2.5 and 31 ± 1.8 min, respectively); furthermore, this strain was incapable of growing in minimal medium supplemented with trimethoprim and thymidine (Table 3).

**Fig 1:**
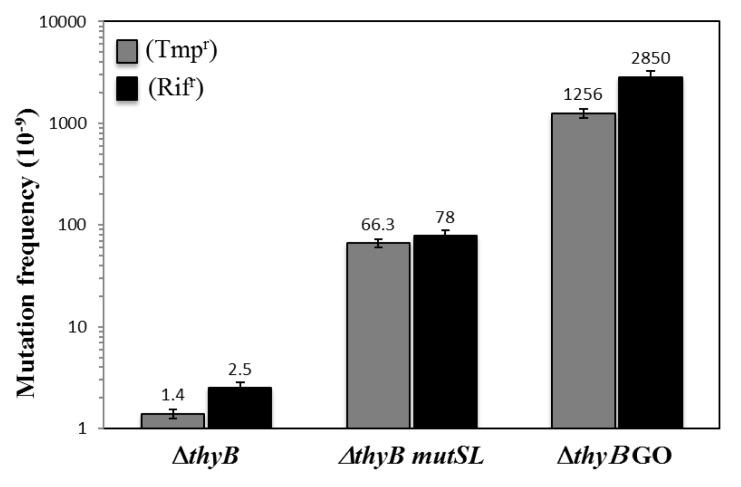
Frequencies of spontaneous mutation to Tmp^r^ (gray bars) and Rif^r^ (black bars) of different *B*. *subtilis* strains. *B*. *subtilis* PERM1037 (Δt*hyB*), PERM1491 (Δ*thyB* GO system deletion), PERM1565 (Δ*thyB mutSL*) were grown overnight in PAB medium, and frequencies of mutation to Rif^r^ or Tmp^r^ were determined as described in Materials and Methods. Each bar represents the mean of data collected from three independent experiments done in sixtuplicate, and the error bars represent standard errors of the means (SEM).

The Δ*thyB* strain was first used to analyze the spontaneous appearance of colonies resistant to trimethoprim in *B*. *subtilis* cells. As shown in Fig 1, colonies with a Tmp^r^ phenotype appeared, in solid minimal medium supplemented with thymidine, with a spontaneous mutation frequency of ~1.4 ± 0.11 × 10^−9^. These results are in good agreement with the mutation frequencies reported for the strain *B*. *subtilis* YB955 using the *rpoB* gene as a marker of mutagenesis [10, 14]. Previous reports, employing the Rif^r^ phenotype demonstrated that inactivation of the canonical mismatch (MMR) and guanine-oxidized (GO) repair systems increased, two and three orders of magnitude, respectively, the spontaneous mutagenesis in growing *B*. *subtilis* cells [8, 9]. As shown in Fig 1, the genetic inactivation of the GO system, which in *B*. *subtilis* is composed of the MutY, MutM and YtkD proteins [9, 38] increased the mutation frequency to Tmp^r^ in the ThyB-deficient *B*. *subtilis* strain around 1000 times. Furthermore, our results revealed that disruption of the *mutSL* operon, encoding the MMR system, increased the frequency of mutation to trimethoprim resistance in the Δ*thyB* strain around 30 times. As shown in Fig 1, the mutation frequencies as determined with the *rpoB* marker in the *mutSL* and GO-deficient strains were similar to those determined with the *thyA* gene. Therefore, the loss-of-function mutagenesis system described in this report is robust and can be successfully employed to study the spectrum of mutagenic events that occur in *thyA* in a population of *B*. *subtilis* cells.

### Mutational spectra of *thyA* in colonies exhibiting a Tmp^r^ phenotype

**Fig 2:**
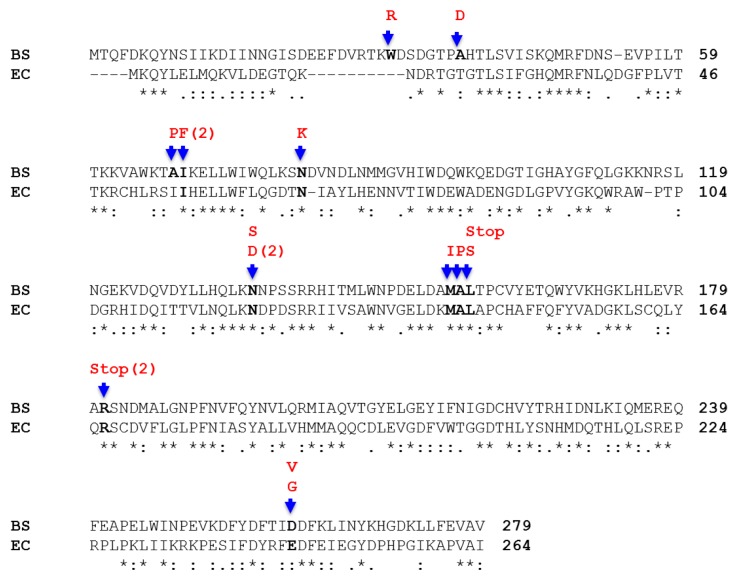
Base substitutions and predicted amino acid changes in the *thyA* ORF from Tmp-resistant *B*. *subtilis* colonies. The deduced amino acid sequences of *thyA* from *B*. *subtilis* and *E*. *coli* were aligned with the Clustal Omega program (http://www.ebi.ac.uk/Tools/msa/clustalo/). The position of the amino acid mutated is indicated with a blue arrow. The amino acid change (in the single letter format) is shown in bold red, above the arrow, the number of changes in a particular position is shown between parentheses. The amino acids involved in the mutation invariably conserved in both proteins are marked in black bold. The mutations that generated a termination codon are indicated as STOP, in bold red letters.

**Fig 3:**
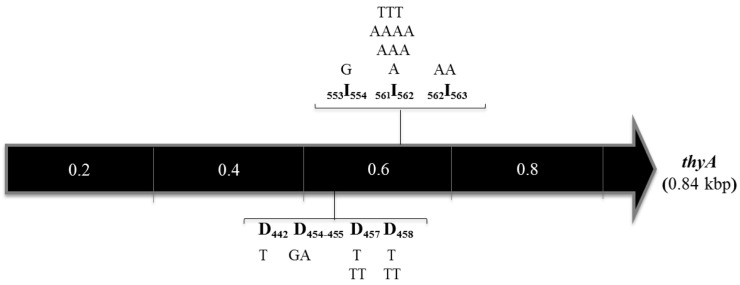
Frame-shift mutations in the *thyA* ORF from *B*. *subtilis* colonies with a Tmp^r^ phenotype. The black arrow depicts the open reading frame of *thyA* in kbp. Nucleotide insertions or deletions at each position are shown above or below the *thyA* ORF, respectively. Oligonucleotide deletions and insertions as well as its positions are shown between brackets. I, insertions; D, deletions.

We further investigated the mutagenic events associated with the loss of TMS function in the *thyB*-deficient strain. It was recently shown, that the heterologous expression of *thy*P3, a homologue of *thyA*, from the ITPG-inducible P*spac* promoter, resulted in the production of mutations in the -5 region of P*spac* conferring trimethoprim resistance in actively growing *B*. *subtilis* cells [39]. In this study, we were interested in identifying mutations affecting the function of ThyA under conditions of native *thyA* expression; therefore, the *thyA* gene of 40 colonies exhibiting a Tmp^r^ phenotype was PCR amplified with high-fidelity DNA polymerase. All the samples produced amplification DNA bands of the expected size for the *thyA* ORF (namely, 837 bp), which were subsequently subjected to DNA sequencing to identify the type of mutations conferring the Tmp^r^ phenotype. A wide spectrum of frameshift mutations and base substitutions were detected in the sequenced *thyA* mutants, predominating the insertions/deletions over the base substitutions in a proportion of 60% to 40% (Table 4 and Figs 2 and 3). Among the substitutions, 63% corresponded to transversions and 37% to transitions events, predominating the A→T (~29%) and A→G (~17%) changes (Table 4). Of note, three of the base changes generated non-sense mutations producing truncated ThyA proteins (Table 4 and Fig 2). It was found that most of the missense mutations in *thyA* resulted in non-conserved amino acid changes, moreover, two of the substitutions changed an alanine residue for the secondary structure-disrupting amino acid proline and one of them switched an isoleucine for the bulky aromatic residue phenylalanine (Table 4 and Fig 2). Importantly, 14 of the 17 missense mutations took place in codons encoding amino acids conserved in both thymidylate synthases (Table 4 and Fig 2). However, additional mutations in residues non-conserved in *E*. *coli* ThyA, were identified in the enzyme from *B*. *subtilis*, including, Ala_68_→Asp, Trp_31_→Arg and Ala_68_→Pro (Fig 2). Thus, in addition to Arg_181_ previously reported as essential for ThyA activity in *E*. *coli* [40], our results identified additional residues necessary for the proper function of ThyA in *B*. *subtilis*.

**Table 4:** Base substitutions in *thyA* alleles from Tmp^r^ colonies.

Position(s) of mutation in the ORF of *thyA* (bp)	Number of Thy^r^ colonies bearing the mutation	Type of mutation	^a^DNA change (Codon involved)	Result of mutation
205	2	Transversion	A → T (69)	Ile → Phe
535	2	Transversion	A → T (179)	Arg → Stop
779	1	Transversion	A → T (260)	Asp → Val
243	1	Transversion	T → A (81)	Asn → Lys
473	1	Transversion	T → A (158)	Leu → Stop
406	2	Transition	A → G (136)	Asn → Asp
407	1	Transition	A → G (136)	Asn → Ser
779	1	Transition	A → G (260)	Asp → Gly
202	1	Transversion	G → C (68)	Ala → Pro
468	1	Transversion	G → C (156)	Met → Ile
469	1	Transversion	G → C (157)	Ala → Pro
91	1	Transition	T → C (31)	Trp → Arg
473	1	Transition	T → C (158)	Leu → Ser
113	1	Transversion	C → A (38)	Ala → Glu

^a^Position of the base substitutions detected in the ORF of *thyA* composed by 279 amino acids. The codon number that was modified by the base change is shown between parentheses.

As noted above, insertion/deletions were also detected in the *thyA* sequence of colonies exhibiting a Tmp^r^ phenotype. As shown in Fig 3, the insertion events took place between the positions 553–562 of the *thyA* ORF and consisted of single base additions, including, 4 adenines 2 thymines or 1 guanine. On the other hand, the frameshift deletions that took place between the positions 455–458 of the *thyA* ORF, involved the single loss of 7 thymines or the dinucleotide guanine-adenine (Fig 3). Notably, the insertion and deletions occurred in the base repeats GGGAAA and GAATT, respectively, and gave rise to truncated non-functional ThyA proteins. Thus, replication errors in these sequences that escaped the action of the mismatch repair machinery may be involved in generating these types of mutations. In support of this notion, the genetic inactivation of the MMR system (MutSL) in the Δ*thyB* strain increased by about two orders of magnitude the mutation frequency to trimethoprim resistance (Fig 1). Altogether, our results revealed that a wide spectrum of mutagenic events may lead to loss of ThyA function corroborating that the Tmp^r^ system informs on the genetic events underlying stress-associated mutagenesis in *B*. *subtilis*.

### Analysis of stationary-phase associated mutagenesis to trimethoprim resistance in nutritionally stressed *B*. *subtilis* cells

**Fig 4:**
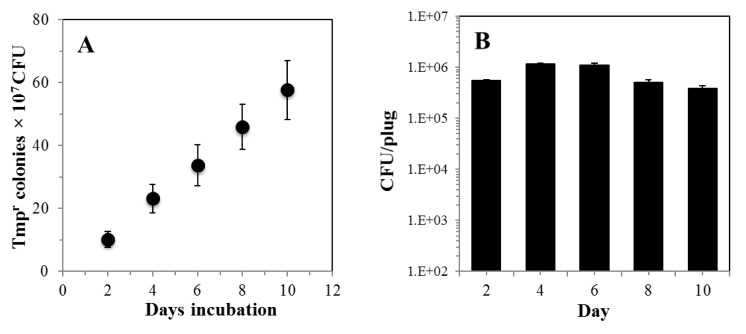
(A). Frequencies of stationary-phase accumulation of Tmp^r^ colonies of strain *B*. *subtilis* PERM1037 (*thyA*^+^ *thyB*^-^) were determined as described in Materials and Methods. Data were normalized to initial cell titers ± SD and represent counts averaged from three separate tests. (B). Ability of strain PERM1037 to survive Met^-^Leu^-^. Data were collected from plugs removed from three plates and titers were plated on media containing all essential amino acids every other day for testing of viability of non-revertant background cells (see Materials and Methods for details). Data is represented as the number of CFU per plug. Error bars represent 1 standard error of the mean.

We employed the Δ*thyB* strain to investigate whether loss-of thymidylate synthase (ThyA) function leading to Tmp^r^ resistance can be employed to measure adaptive mutagenesis in *B*. *subtilis*. To this end, *B*. *subtilis* cells of the ThyB-deficient strain collected from the stationary-phase of growth were extensively washed to eliminate residual nutrients and plated in selective Spizizen Minimal Medium (SMM) as described in Materials and Methods. The number of colonies that acquired a Tmp^r^ phenotype under growth-limited conditions was scored every two days for a ten days period. The results revealed that Tmp^r^ colonies from the Δ*thyB thyA*^+^ accumulated over a period of ten days with a frequency of ~65 ± 11 × 10^−7^ (Fig 4A), demonstrating the feasibility of employing this loss-of-function system to study mutagenesis in non-growing *B*. *subtilis* cells. Notably, the increase in the number of Tmp^r^ colonies took place without significant changes in the viable cell count number providing thus additional support for this assumption (Fig 4B).

### Transcription-associated mutagenesis analysis in growth-limited *B*. *subtilis* cells acquiring a Tmp^r^ phenotype

Previous studies have reported on the role of transcription in stationary-phase mutagenesis in *B*. *subtilis* [12]. It has been shown that a combination of transcriptional derepression and error-prone repair events in stress conditions can modulate the generation of mutations in highly transcribed DNA regions [13, 41].

**Fig 5:**
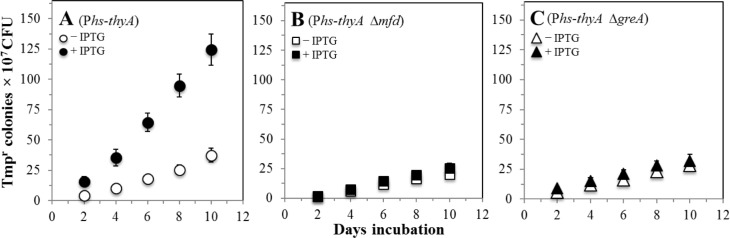
Frequencies of stationary-phase accumulation of Tmp^r^ colonies of strains *B*. *subtilis* PERM1100 (A), PERM1171 (B) and PERM1192 (C) under repressed (–IPTG; white symbols) or induced (+ IPTG: black symbols) were determined as described in Materials and Methods. Data represent counts from three plates averaged from three separate tests, normalized to initial cell titers.

To investigate whether acquisition of Tmp^r^ resistance can be used to study transcription-associated mutagenesis (TAM) in nutritionally stressed bacteria we engineered a double *thyA thyB* knock out strain that overexpressed a wild type copy of *thyA* from the IPTG-inducible Phyperspank (P*hs*) promoter; this strain was termed *B*. *subtilis* PERM1100 (Table 1). As specified in Table 3, the *thyA thyB* mutant was able to grow in SMM supplemented with Tmp and thymidine. However, IPTG-induction of *thyA* expression abrogated the growth of the strain PERM1100 in this medium. Further, the lack of two amino acids (methionine and leucine) in the incubation agar media prevented the growth of cells. Therefore, the strain's properties and the double amino acid starvation allowed us to inquire whether derepression of *thyA* in growth-limited cells promote mutagenic events influencing the production of Tmp^r^ colonies. As shown in Fig 5A in reference to the non-induced condition, derepression of the P*hs*-*thyA* construct resulted in a 3-fold increase in the production of colonies with a Tmp^r^ phenotype.

**Fig 6:**
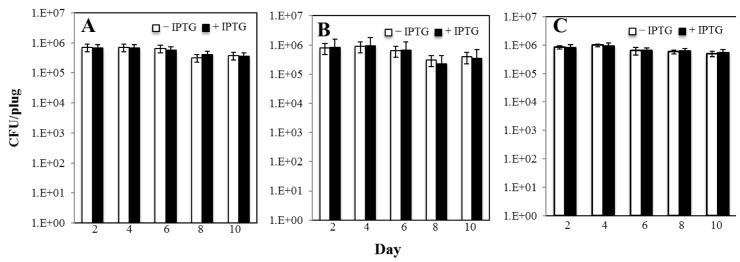
Ability of strain *B*. *subtilis* PERM1100 (A), PERM1171 (B) and PERM1192 (C) to survive Met^-^Leu^-^ starvation in selective media supplemented (black bars) or not (white bars) with IPTG. Data were collected from plugs removed from three plates and titers were plated on media containing all essential amino acids every other day for testing of viability of non-revertant background cells (see Materials and Methods for details). Data is represented as the number of CFU per plug. Error bars represent 1 standard error of the mean.

Our loss-of function mutagenesis system indicate that increased transcription levels of *thyA* correlated with an increased production of colonies with a Tmp^r^ phenotype in the Δ*thyAthyB amyE*::P*hs*-*thyA* strain. Of note, these results are in good agreement with former studies showing a positive correlation between derepression of a *leuC* allele and production of Leu^+^ prototrophs in *B*. *subtilis* [13]. Furthermore, viability of the strain PERM1100 did not significantly change in the absence and/or presence of the inducer IPTG (Fig 6A); therefore, the increase in the number of Tmp^r^ can be uncoupled from the growth of the sensitive strain in the plate. To confirm that accumulation of Tmp^r^ mutants were due to increases in transcription levels of *thyA*, the mRNAs of this gene were quantified by qRT-PCR in stationary-phase cells of strain PERM1100 under induced and non-induced conditions. The results of this analysis showed that the mRNA levels of *thyA* increased over 3-fold (i.e. from 0.5 ± 0.06 to 4.5 ± 0.4) when IPTG was added to the medium. Therefore, the loss-of-function system implemented here can be successfully employed to investigate the mutagenic processes occurring in transcriptionally active DNA regions that allow cells to escape from non-proliferating conditions. Of note, the discovery of missense and nonsense mutations affecting *B*. *subtilis* ThyA function (Fig 2), will allow to adapt this system to determine the production of *thyA*^+^ revertants in nutritionally stressed *thyA*^-^ *thyB*^-^ cells overexpressing point-mutated alleles of *thyA*.

A recent study revealed that Mfd, a protein that couples DNA repair with transcription, promotes mutagenic events that increase the production of His^+^, Met^+^ and Leu^+^ revertants in starved cells of strain *B*. *subtilis* YB955 [12, 13]. Here, we inquired whether the Mfd requirement for transcription-associated SPM can be detected with the loss-of-function Tmp^r^ mutagenesis system. To this end, the gene *mfd* was genetically inactivated in the Δ*thyA thyB* strain bearing the P*hs-thyA* construct. The number of Tmp^r^ colonies produced by this strain under starving conditions was determined over a period of ten days under conditions where the *thyA* gene was repressed or derepressed for transcription. As shown in Fig 5B, in reference to the strain harboring an intact copy of Mfd, the number of adaptive Tmp^r^ colonies produced by the Mfd-deficient strain did not significantly differ under conditions of repression or derepression for the *thyA* gene. Therefore, employing a system that overexpresses a different gene; namely, *thyA*, our results provide further support for the concept that Mfd is a factor required for TMM in growth-limited *B*. *subilis* cells. As noted above, NusA, the elongation factor of the RNA polymerase has been found to be necessary for stress-induced mutagenesis in *Escherichia coli* [16]. Remarkably, disruption of *greA* in the *thyA thyB* strain abrogated the production of Tmp^r^ colonies under conditions that induced *thyA* expression. Thus, as shown in Fig 5C, the levels of Tmp^r^ mutagenesis in this strain were almost similar under conditions that induced or repressed *thyA*. Importantly, these results took place in the absence of growth since viability of the GreA-deficient strain did not significantly change in the absence and/or presence of the inducer IPTG (Fig 6C). Based on these results is feasible to speculate that in nutritionally stressed *B*. *subtilis* cells, processing of paused or backtracked RNAP-DNA complexes promote mutagenic events that allow cells to escape from growth-limited conditions. Interestingly, in *E*. *coli*, both Mfd and GreA are speculated to prevent UvrD-dependent RNAP backtracking and repair during transcription [42]. However, the consequences of such transcriptional events on growth and stress-associated mutagenesis remain to be elucidated.

In summary, the mutagenesis method implemented here and the contribution of GreA to TMM in *B*. *subtilis* cells add further elements to our understanding on how bacteria develop beneficial mutations, including antibiotic resistance, under stressful conditions.
